# Clonal and Horizontal Transmission of *bla*_NDM_ among Klebsiella pneumoniae in Children’s Intensive Care Units

**DOI:** 10.1128/spectrum.01574-21

**Published:** 2022-06-27

**Authors:** Bo Fu, Dandan Yin, Chengtao Sun, Yingbo Shen, Dejun Liu, Rina Bai, Rong Zhang, Jianzhong Shen, Fupin Hu, Yang Wang

**Affiliations:** a Beijing Key Laboratory of Detection Technology for Animal-Derived Food Safety, College of Veterinary Medicine, China Agricultural University, Beijing, People’s Republic of China; b Institute of Antibiotics, Huashan Hospital, Fudan University, Shanghai, China; c Key Laboratory of Clinical Pharmacology of Antibiotics, Ministry of Health, Shanghai, China; d CAS Key Laboratory of Pathogenic Microbiology and Immunology, Institute of Microbiology, Chinese Academy of Sciences, Beijing, China; e The Second Affiliated Hospital of Zhejiang University, Zhejiang University, Hangzhou, China; Memorial Sloan Kettering Cancer Center

**Keywords:** carbapenem, *bla*_NDM_, *Klebsiella pneumoniae*, children, intensive care unit

## Abstract

Increasing infections caused by *bla*_NDM_-carrying Klebsiella pneumoniae (NDM-KP) are an urgent threat to children with weakened immunity and limited antibiotic use. Preventing and intervening in NDM-KP infections requires a clear understanding of the pathogen’s molecular and epidemiological characteristics. We investigated the prevalence and characteristics of NDM-KP in six children’s hospitals from five Chinese provinces/municipalities. We collected 111 NDM-KP strains (40 NDM-1, one NDM-4 and 70 NDM-5) from neonatal intensive care units (NICUs) and pediatric intensive care units (PICUs) from June 2017 to June 2018; these strains accounted for 31.62% of all carbapenem-resistant K. pneumoniae (CR-KP). Although NDM-KP isolates exhibited high resistance to all carbapenems, including ertapenem (MIC: ≥32 mg/L, 96.4%), imipenem (MIC: ≥16 mg/L, 90.1%) and meropenem (MIC: ≥16 mg/L, 99.1%), they were fully sensitive to amikacin, tigecycline and polymyxin B, and presented low resistance to levofloxacin (9.9%) and gentamicin (15.3%). Whole-genome sequencing was conducted to gain insight into the molecular characterizations of NDM-KP isolates. The NDM-KP isolates belonged to 20 sequence types (STs), and ST2407 (*n* = 45) dominated in one hospital from Chengdu. ST2407 isolates with fewer single-nucleotide polymorphisms (SNP < 38) were found either in the same hospital or different hospitals. Most *bla*_NDM_ (81.1%, 90/111), including all *bla*_NDM-5_ and *bla*_NDM-4_ and 47.5% (19/40) of *bla*_NDM-1_, in NDM-KP isolates with 13 STs were associated with the IncX3 plasmid. Our results indicated that both explosive clonal transmission and horizontal transmission of *bla*_NDM_ occur among NDM-KP strains in children's hospitals. These data provide a basis for preventing and controlling NDM-KP-associated infectious diseases in hospitalized children, especially in neonates.

**IMPORTANCE** The *bla*_NDM_ gene is playing an increasingly important role in infections caused by CR-KP, especially in children. However, systematic detection and bioinformatics analysis of NDM-KP in children's hospitals are lacking in China. In this study, a total of 111 NDM-positive K. pneumoniae isolates were selected from the China Antimicrobial Surveillance Network for further investigation. The isolates were further characterized using state-of-the-art molecular techniques. Our findings suggested the clonal and horizontal transmission of *bla*_NDM_ in K. pneumoniae in NICUs/PICUs. Key plasmids (IncX3) and ST diversity contribute to the spread of *bla*_NDM_. In addition, our findings provided recommendations for pediatric clinicians to use antibiotics to treat NDM-KP infections. Our current large-scale epidemiological survey would support further infection intervention strategies of NDM-KP in NICU/PICU of children's hospitals.

## INTRODUCTION

Klebsiella pneumoniae is one of the most common and important pathogens causing community-acquired and hospital-acquired infectious bacterial diseases. Particularly in hospital-acquired infections, K. pneumoniae causes various infectious diseases, including sepsis, urinary tract infections, pneumonia and soft tissue infections, especially in immunocompromised hosts such as newborns (1). The emergence and rapid spread of carbapenem-resistant K. pneumoniae (CR-KP) in recent decades led to failed infection treatments, making it an urgent threat to global public health (2). The World Health Organization listed carbapenem-resistant Enterobacterales, including CR-KP, as priority drug-resistant bacteria requiring novel treatment (3). Although it emerged later than other carbapenem resistance genes, such as *bla*_KPC_, *bla*_IMP_, *bla*_VIM_ and *bla*_OXA-48_ (4), the *bla*_NDM_ gene has been rapidly identified in clinical K. pneumoniae isolates worldwide (5–8).

The situation in China is grim as well. Data from the China Antimicrobial Surveillance Network (CHINET) revealed that CR-KP increased from 2.9% in 2005 to 27.1% in 2021 (http://www.chinets.com/). In addition to *bla*_KPC_-carrying K. pneumoniae (KPC-KP) (9), NDM-KP has begun to enter neonatal intensive care units (NICUs) and pediatric intensive care units (PICUs), with a handful of outbreaks among child patients in many areas of China (10–15). Notably, the isolation rate of NDM-KP in children's hospitals is increasing annually (14, 16). Considering the limited antibiotic selection for children, the characteristics of NDM-KP isolated from children need to be investigated.

Previous reports described NDM-KP outbreaks based mostly on fragmented surveillance of individual children’s hospitals and lacked systematic comparisons of the molecular epidemiology and strain characteristics between hospitals. To elucidate the epidemiology and molecular characteristics of NDM-KP in the NICUs/PICUs of different children’s hospitals, we collected NDM-KP clinical isolates from six children’s hospitals from five Chinese provinces or municipalities for 1 year (June 2017 to June 2018). Whole-genome sequencing and analysis were undertaken to gain insight into characterizing the isolates.

## RESULTS

### Phenotypes and genotypes of NDM-KP in six children’s hospitals.

**FIG 1:**
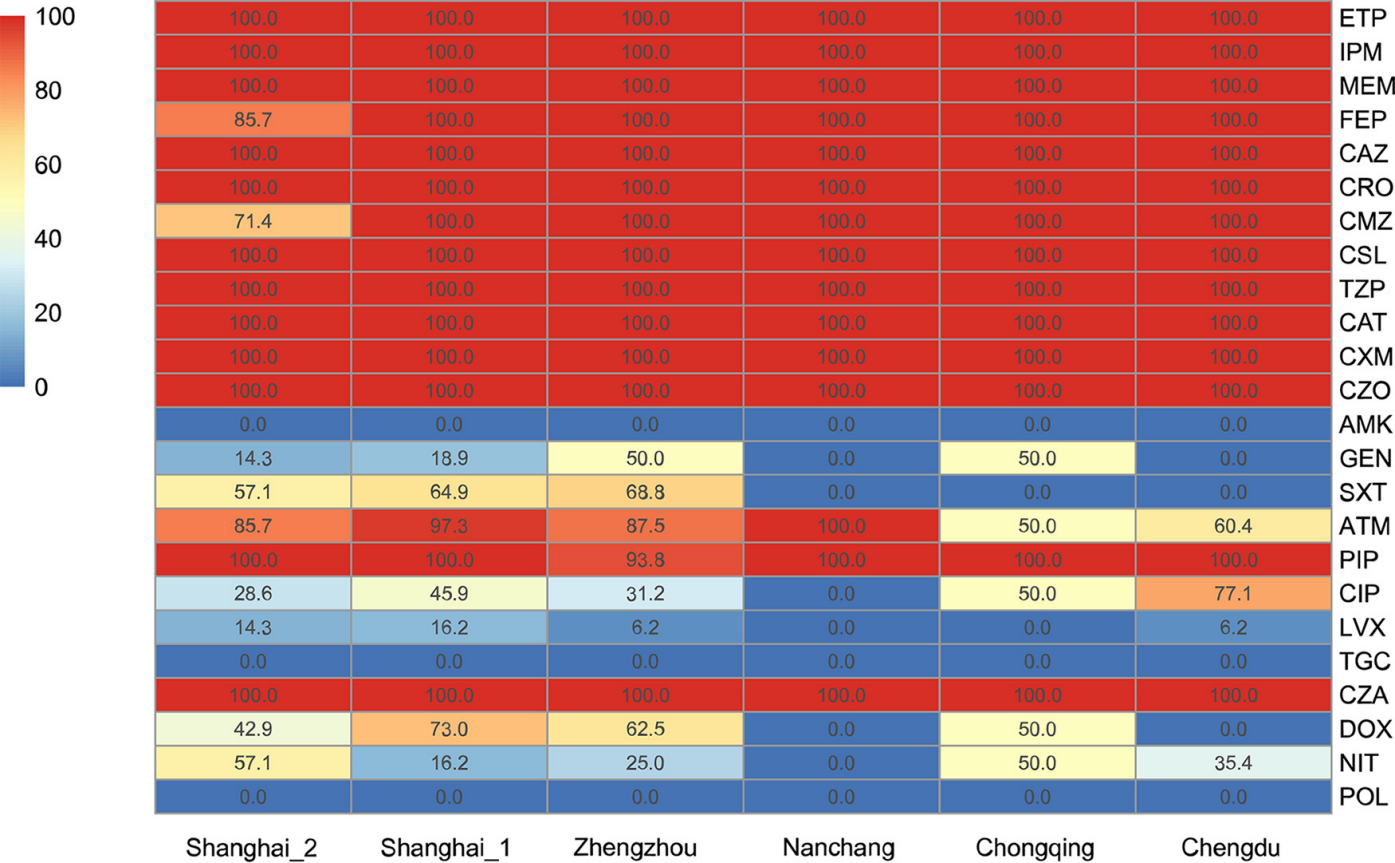
Antimicrobial-resistance profiles of 24 antimicrobial agents against NDM-KP. The number represents the antimicrobial resistance rate of the NDM-KP isolates. ETP: ertapenem; IPM: imipenem; MEM: meropenem; FEP: cefepime; CAZ: ceftazidime; CRO: ceftriaxone; CMZ: cefmetazole; CSL: cefoperazone-sulbactam; TZP: piperacillin-tazobactam, CAT: ceftolozane-tazobactam; CXM: cefuroxime; CZO: cefazolin; AMK: amikacin; GEN: gentamicin; SXT: trimethoprim-sulfamethoxazole; ATM: aztreonam; PIP: piperacillin; CIP: ciprofloxacin; LVX: levofloxacin; TGC: tigecyclin; CZA: ceftazidime-avibactam; DOX: doxycycline; NIT: nitrofurantoin; POL: polymyxin B.

The *bla*_NDM_ gene was detected in 111 CR-KP isolates originating from six children’s hospitals and accounted for 31.62% (111/351, 95% confidence interval [CI]: 26.8%–36.8%) of all CR-KP isolates in Chengdu (48/49, 97.96%), Chongqing (2/34, 5.88%), Nanchang (1/5, 20%), Zhengzhou (16/130, 12.31%), Shanghai_1 (37/111, 33.33%), and Shanghai_2 (7/22, 31.82%). All CR-KP isolates showed high resistance to carbapenems, including ertapenem (MIC: 96.4%, ≥32 mg/L), imipenem (MIC: 90.1%, ≥16 mg/L) and meropenem (MIC: 99.1%, ≥16 mg/L; Fig. 1). These isolates also exhibited high resistance rates to ceftazidime-avibactam (100%), piperacillin-tazobactam (100%), cefoperazone-sulbactam (100%), ceftolozane-tazobactam (100%), and other β-lactams (piperacillin, cefepime, ceftazidime, ceftriaxone, cefmetazole, cefuroxime, and cefazolin; 98.2%–100%); moderate resistance to doxycycline (*n* = 42; 37.8%), ciprofloxacin (*n* = 62; 55.9%) and aztreonam (*n* = 87; 78.4%); low resistance to gentamicin (*n* = 17; 15.3%), trimethoprim-sulfamethoxazole (*n* = 39; 35.1%), nitrofurantoin (*n* = 32; 28.8%) and levofloxacin (*n* = 11; 9.9%); and sensitivity to amikacin, tigecycline and polymyxin B (Fig. 1). No strains showed the hypermucoviscous phenotype.

**FIG 2:**
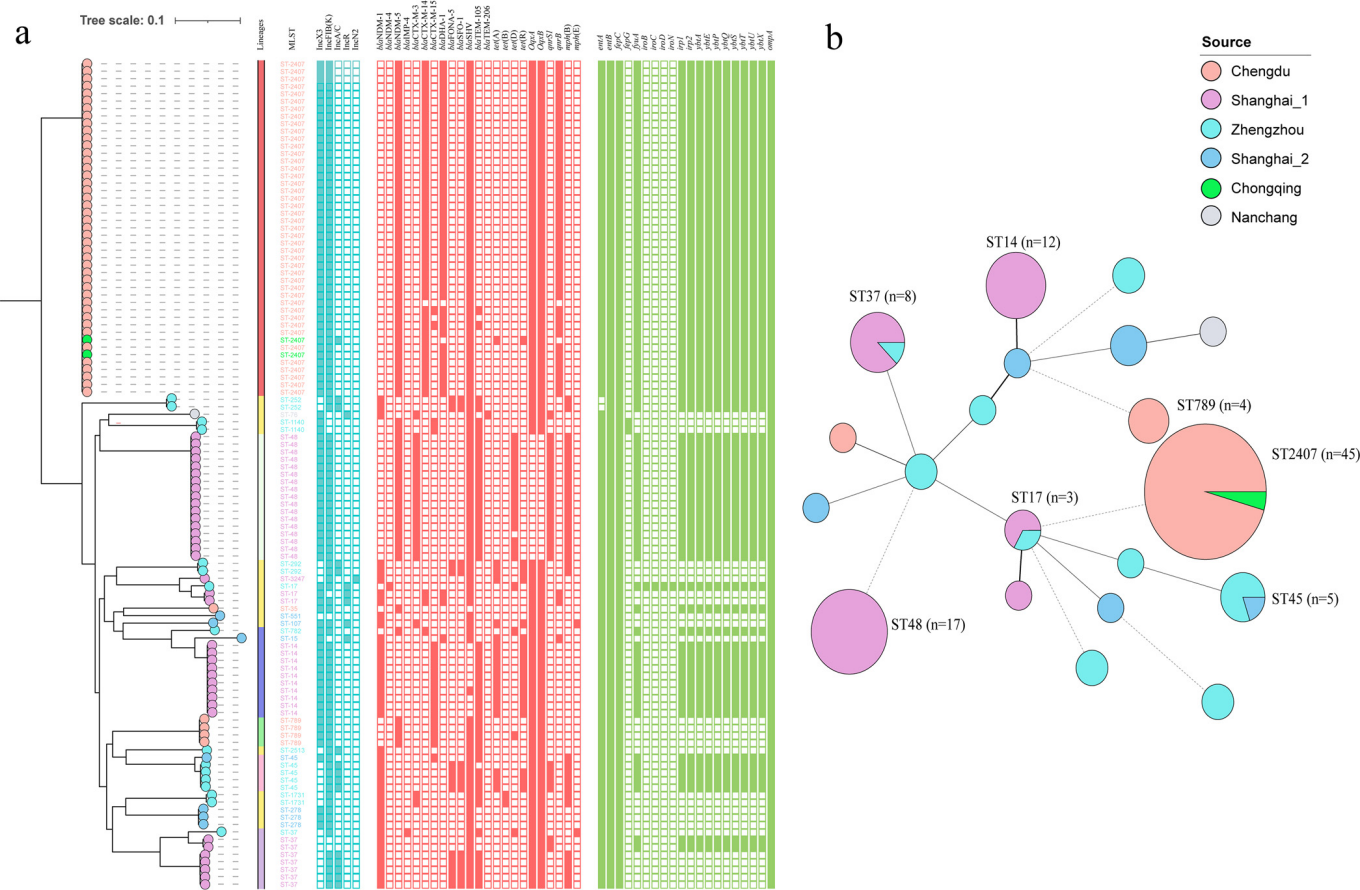
Population structure of NDM-KP isolates. (a) Phylogeny of core-genome SNPs in 111 NDM-KP isolates. K. pneumoniae ST2407 S6, sequenced on MinION, was used as a reference strain. Sources of the isolates are differentiated by six colors. Inc-type plasmid (bule square), antimicrobial-resistance genes (red square), and virulence-associated genes (green square) among the isolates are denoted by filled squares for presence and empty squares for absence. (b) Minimum-spanning tree of NDM-KP isolates based on a core-genome MLST (cgMLST).

Antibiotic-resistance and virulence-associated genes in the 111 NDM-KP isolates were identified from WGS data (Fig. 2A, Table S1). Seventy NDM-KP (63%) harbored *bla*_NDM-5_, 40 (36%) harbored *bla*_NDM-1_, and one (1%) harbored *bla*_NDM-4_. One ST37 strain (A96) carried two CR genes, *bla*_NDM-1_ and *bla*_IMP-4_. Some NDM-KP isolates also harbored the ESBL genes, *bla*_CTX-M-14_ (47; 42.3%), *bla*_CTX-M-15_ (19; 17.2%), *bla*_CTX-M-3_ (21; 18.9%) and *bla*_SFO-1_ (14; 12.6%); the tetracycline-resistance genes, *tet*(A) (19; 17.1%), *tet*(B) (2; 1.8%) and *tet*(D) (20; 18%); and the fluroquinolone-resistance genes, *oqxAB* (94; 84.7%), *qnrB* (46; 41.4%) and *qnrS1* (24; 21.6%). For the genes associated with hypervirulent K. pneumoniae, the categorical virulence scores ranged from 0–5; 24.3% (27/111) of the K. pneumoniae isolates scored 0, and the remaining K. pneumoniae (75.7%, 84/111) isolates scored 1, suggesting that the NDM-KP isolates carried few genes mediating high virulence. Strains scoring 1 carried the siderophore yersiniabactin virulence loci. Only one strain, A156, carried the hypermucoid locus, *rmpA*, and the salmochelin loci, *iro*B, *iro*C, *iro*D and *iro*N. Additionally, 34.2% (38/111) of the K. pneumoniae isolates had different degrees of deletions in the outer membrane protein, OmpK35.

### Nosocomial *bla_NDM_* outbreak and transmission caused by K. pneumoniae diversity.

The 111 NDM-KP isolates were assigned to 20 MLST types (Table S1). The minimum-spanning tree indicated the clonal transmission characteristics of some NDM-KP isolates in the NICUs/PICUs (Fig. 2B). For example, clone transmission of ST2407 (*n* = 45) occurred in a children's hospital from Chengdu. The same ST, like ST2407, ST37, ST45, and ST17, could exist in two children's hospitals that were geographically far apart. Additionally, 10 and nine STs occurred in Shanghai (two hospitals) and Zhengzhou (one hospital in Henan), respectively. Like the STs, the NDM-KP serotypes also exhibited diverse profiles. Identification of capsule synthesis loci revealed 20 different types. Each ST, except for ST37, corresponded to only one capsule synthesis locus. ST37 had three capsular synthesis loci: K8, K15 and K38. All K. pneumoniae ST14 serotypes were K2, which has been associated with high pathogenicity. Five O antigen (LPS) serotypes (O1–O5) were predicted (Table S1). The data showed that *bla*_NDM_ can exist in various K. pneumoniae strains, showing its widespread spread at cloning levels in children's hospitals.

### Phylogenetics of NDM-KP in six children's hospitals.

Phylogenetic analysis of the core-genome single-nucleotide polymorphisms (SNPs) revealed seven distinct population structure lineages among the 111 NDM-KP isolates (Fig. 2A). Shallow branching and scattered population structure also occurred: the same K. pneumoniae STs (except ST37) in the same hospital exhibited little branching and few SNPs (5–38), suggesting an outbreak of NDM-KP in one hospital. Notably, all NDM-KP ST2407 isolates were clustered as the significant phylogroup with limited SNP (5–29) divergences. The core genomes of two isolates from Chongqing and 43 isolates from Chengdu were nearly identical, with <25 SNPs (Table S2). These two hospitals are geographically distant (straight-line distance >280 km), indicating the spread of *bla*_NDM_ through clonal transfer. In contrast, on the three branches for ST17, ST37, and ST45, the core genomes of the same STs from two children's hospitals differed significantly.

### Epidemiology of the *bla*_NDM_-IncX3 plasmid in children's hospitals.

**FIG 3:**
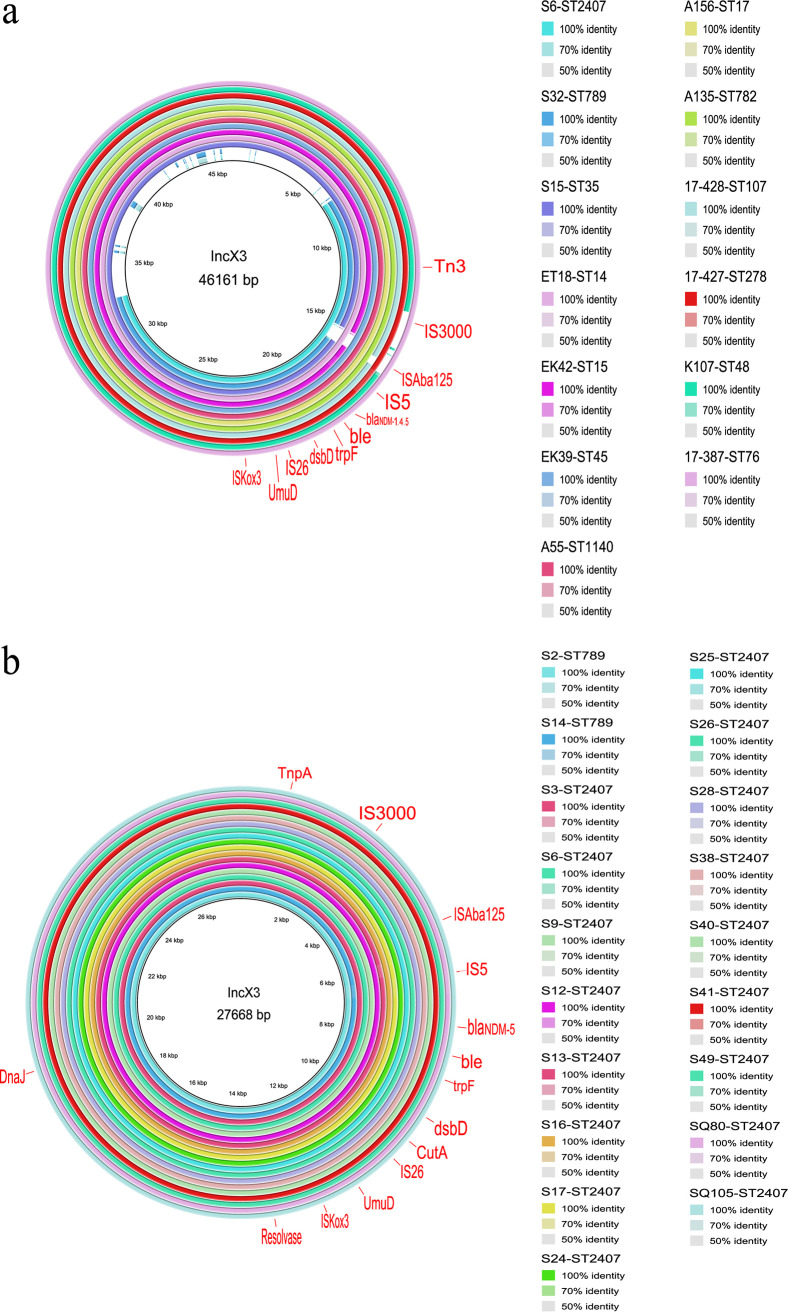
Schematic diagram of mobile genetic elements integrated in NDM-KP isolates. (a) IncX3 plasmid carrying *bla*_NDM_ was present in 13 K. pneumoniae STs. (b) Evolved IncX3 plasmid carrying *bla*_NDM_ in ST789 and ST2407 K. pneumoniae.

Plasmids are likely the most common carriers for *bla*_NDM_ gene (17). WGS data detected major Inc-type plasmids, including IncX3, IncFIB(K), Inc(A/C), IncR and IncN2. A complete high prevalence 46.1 kb IncX3 plasmid (GenBank accession number: CP049352) carrying *bla*_NDM-5_ isolated from E. coli in China was used as a reference, then the BLAST Ring Image Generator (BRIG) was used to compare it with the WGS of different strains, revealing that the *bla*_NDM_-IncX3 plasmid presented in 13 different NDM-KP STs (Fig. 3A) and in 81.1% of the total NDM-KP (90/111), which is highly homologous to the reference plasmid. By BLAST analysis of larger contigs (>34 kb), we identified 18 isolates carrying the *bla*_NDM_-IncX3 plasmid. The Bandage association analysis revealed a high probability connection between IncX3 plasmids and *bla*_NDM_ in the remaining 72 isolates. However, circularization of plasmids (i.e., use of Illumina plus Nanopore) in these isolates would prove this suggestion. IncX3 plasmid presented in all *bla*_NDM-4_ and *bla*_NDM-5_ isolates and 45.7% (19/40) of *bla*_NDM-1_ isolates, and this plasmid could be identified from isolates of the same lineage or different lineages in the same hospital, suggesting the spread of *bla*_NDM_ through horizontal transfer (Fig. 2A). Notably, BRIG analysis showed that 49 IncX3 plasmids carried by ST2407 (*n* = 45) and ST789 (*n* = 4) isolates from the Sichuan and Chongqing hospitals was smaller than the original IncX3 plasmid (46.1 kb), and the conjugation-associated type IV secretion system (T4SS) play as the missing part. Therefore, we randomly selected one ST2407 isolate S6 carrying the smaller IncX3 plasmid for MinIon sequencing and confirmed the 27.7 kb size of the plasmid with T4SS deletion (Table S3). Further, the IncX3 plasmids in these NDM-KP ST2407 (*n* = 17) and ST789 (*n* = 2) strains had higher homology with this 27.7 kb IncX3 plasmid (Fig. 3B).

### Mobile genetic element arrangements in spreading *bla*_NDM_ in children's hospitals.

**FIG 4:**
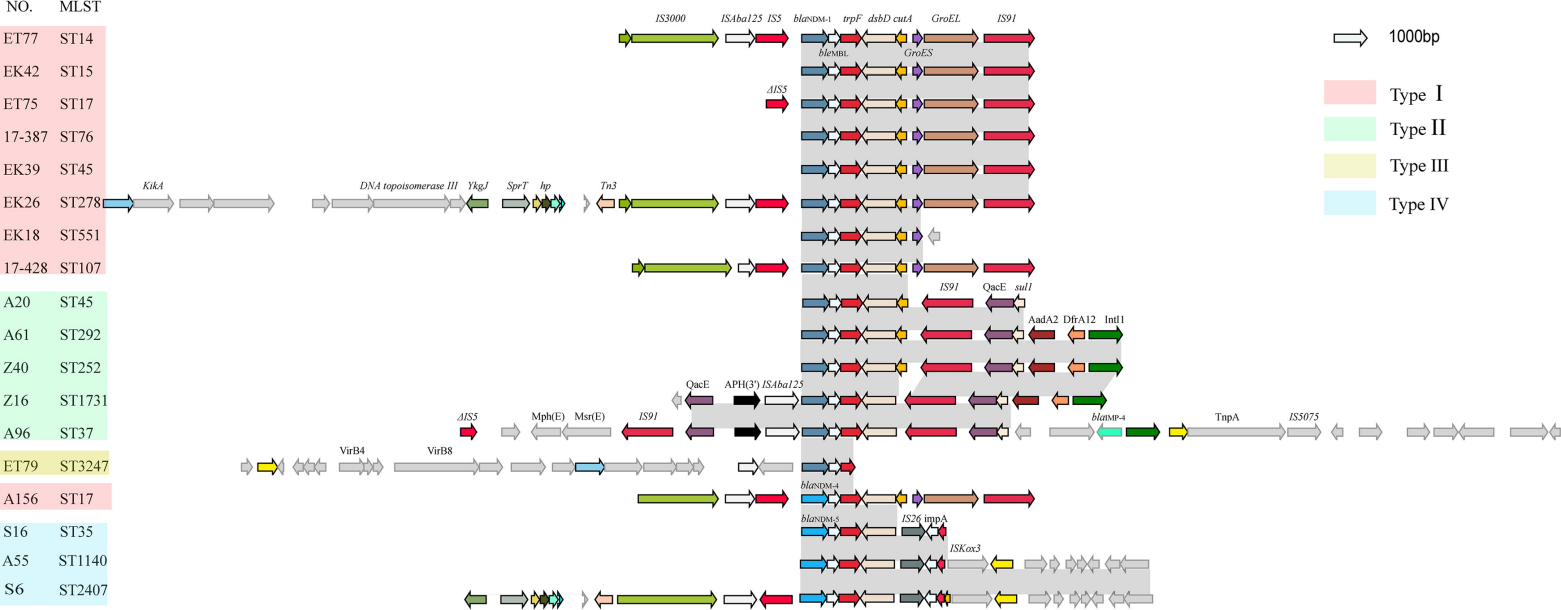
Genetic environment of *bla*_NDM_ in K. pneumoniae. The four different types are represented by four colors. Open reading frames are designated by arrows indicating the direction of transcription and colored based on their predicted gene functions. Dark gray shading indicates homologous regions.

To further analyze the transfer of *bla*_NDM_ via mobile genetic elements (MGEs), comparison of the *bla*_NDM_ genetic environment in all 20 NDM-KP STs illustrated a conservative and complex combination of multiple genetic vehicles in spreading *bla*_NDM_ (Fig. S2 and Fig. 4). The genetic environments of *bla*_NDM_ were clustered into four types according to homologous regions, ST and *bla*_NDM_ genotypes, showing a relatively conserved architecture in the variable context surrounding *bla*_NDM_. All four types retained the conserved sequence *bla*_NDM_-*ble*_MBL_-*trpF*-*dsbD.* All *bla*_NDM-5_ belonged to type 4 (*n* = 70), *bla*_NDM-4_ to type 1 (*n* = 1), and *bla*_NDM-1_ to types 1 (*n* = 19), 2 (*n* = 20) and 3 (*n* = 1). Compared with the conservative flanking region of *bla*_NDM-5_, the surrounding environments of *bla*_NDM-1_ exhibited more diverse, likely owing to the different Inc types of plasmids carrying *bla*_NDM-1_. Both types 1 and 4 were associated with the IncX3 backbone, with differences in the heat shock protein-related GroES and GroEL, which only appear downstream of *bla*_NDM_ in type 1. The upstream IS*3000*, IS*5*, and the downstream IS*91* of the segment *bla*_NDM_-*ble*_MBL_-*trpF*-*dsbD* are conserved. Type 2 belonged to the plasmid backbone of IncFIB(K) (contigs > 137 kb), in which the strain A96 of ST37 coexisted with *bla*_IMP-4_ at the 8921-bp downstream position of *bla*_NDM-1_. Additionally, the sulfanilamide-resistance gene, *sul1*, also coexisted with the downstream *bla*_NDM-1_. The *bla*_NDM-1_-carrying type 3 belonged to the plasmid backbone of IncN2 (contigs > 41 kb), in which the upstream *virB4* and *virB8* were associated with plasmid conjugation transfer, suggesting that *bla*_NDM-1_ can be transferred horizontally by plasmids other than IncX3.

## DISCUSSION

Clinical CR-KP in China is an increasing concern, and its prevalence is higher in children than in adults (China Antimicrobial Resistance Surveillance System, http://www.carss.cn/). Compared with KPC-KP, the notorious pathogen dominant in both children's and adult hospitals, NDM-KP has caused a higher proportion of CR-KP in children’s hospitals than in adult hospitals in recent years (18). As the possible transmission link for NDM-E. coli between humans and animals has been established (19–21), we proposed that the increasing trend for NDM-KP in child patients in this study might be associated with animals for the following four reasons. First, different from the ST11-dominant KPC-KP in China (9, 22, 23), the NDM-KP isolates collected from children’s hospitals exhibited diverse profiles (20 STs) and were not limited to one superior clone. Most clones, including ST45, ST48, and ST37, have been detected and are being transferred in livestock and poultry production chain (24–26). Second, the *bla*_NDM_-IncX3 dominant in children NDM-KP was proved to be a successful plasmid among various commercial farm animals (pigs and chicken), backyard animals (pigs, chickens, cattle, pets), and other animals (flies and birds). The high conjugation transfer frequency (27), low fitness cost (28) and favorable stability of the IncX3 plasmid in many Enterobacteriaceae may facilitate the circulation of *bla*_NDM_-IncX3 plasmids in bacteria of both animal and human origin. Third, unlike *bla*_KPC_, which disseminates via stable association with a lineage of K. pneumoniae, the spread of *bla*_NDM_ is mediated by transient associations of diverse plasmids (including IncX3) with multiple lineages of K. pneumoniae (17). Notably, *bla*_NDM_, but not *bla*_KPC_, was frequently detected in high abundances in farm-animal feces and in high prevalence in bacteria of farm-animal origin (29). Finally, children are generally considered more susceptible to infection because of the immaturity of their immune and intestinal systems (30). CR-KP can emerge from the intestinal lumen and invade the bloodstream of vulnerable patients, causing disseminated infection (31). Thus, NDM-KP may be transmitted directly (via the food chain) or indirectly (in the environment) from animals to children, leading to colonization and infection. Therefore, the increasing NDM-KP in children’s hospitals suggests that the patterns and extent of the impact of non-human factors on NDM-KP infections in children must be investigated.

Our results for both antimicrobial-resistance genes and phenotypes indicated the presence of multidrug-resistant (MDR) K. pneumoniae in children's hospitals. The use of ß-lactams can co-select resistance determinants for different antibiotics harbored in the same plasmids as *bla*_NDM_, such as sulfonamides and even quinolones, which are not used in common children's treatment. These MGEs likely contribute to the prevalence of *bla*_NDM_ and increase the threat to children’s health with further limited spectrum antibiotics. Fortunately, all NDM-KP are completely sensitive to amikacin, tigecycline and polymyxin B and highly sensitive to levofloxacin and gentamicin, which helps fight against NDM-KP. Gentamicin and polymyxin B are more suitable for clinical children, while amikacin (32), levofloxacin (33, 34) and tigecycline (35) are used with caution due to unclear safety concerns. Clinicians may consider these antibiotics as alternative options for treating NDM-KP infections. Additionally, these MDR K. pneumoniae are not highly virulent, which thus reduces the risk of severe infections in children. Nevertheless, these virulence-related genes and plasmids should not be ignored. Previous studies have predicted that the rate at which MDR K. pneumoniae acquires virulence plasmids far exceeds the rate at which hypervirulent K. pneumoniae (36) acquires MDR plasmids.

This work had several limitations. First, the amount of data collected from the six hospitals was inconsistent, which may have led to bias in the analysis. Second, we only collected the strain characteristic information during the study, and the individual medical history information (e.g., demographics [sex and age], antibiotics exposure, disease types) of the patients that the strains were isolated from was mismatched or absent due to the improper storage, thus we could not conduct a correlational study between epidemiological data and strain genetic information. Finally, we have not determined the mechanism causing the increased NDM-KP in children and its relevance to NDM-KP in animals; this will be a focus in our subsequent studies.

Our study revealed the clonal and horizontal transmission of *bla*_NDM_ in K. pneumoniae in NICUs/PICUs. Key plasmids (IncX3) and ST diversity accelerate the spread of *bla*_NDM_. Considering these results, clinicians should include screening for NDM-KP when diagnosing infectious diseases in children, especially newborns, to avoid treatment time delays and antibiotic failure. Monitoring and data collection on *bla*_NDM_ in children should be strengthened to better understand its epidemic patterns. Our results also suggest that there may be more confounding factors for prevalence of *bla*_NDM_ in children, therefore, the “One Health” perspective is needed to address this increasing threat.

## MATERIALS AND METHODS

### Bacterial isolation and identification.

We collected 351 nonduplicate CR-KP clinical isolates from six children's hospitals in five provinces or municipalities across China (member units of the CHINET Study Group; Fig. S1) from June 2017 to June 2018. All CR-KP strains were identified using Vitek matrix-assisted laser desorption ionization-time of flight mass spectrometry (MALDI-TOF MS, bioMérieux, Marcyl’Étoile, France) and selected by the Kirby-Bauer antimicrobial susceptibility method during the routine daily work of the microbiology laboratory in each children hospital. PCR (37) was used to screen the target *bla*_NDM_-positive strains for follow-up study. The NDM-KP isolates were obtained from sputum (*n* = 91), bronchoalveolar lavage fluid (*n* = 7), urine (*n* = 2) and blood (*n* = 11), collected from the NICU (*n* = 83) or PICU (*n* = 28) at six children's hospitals (Table S1).

### Antimicrobial susceptibility testing.

The MICs for 24 antimicrobial agents were determined for all *bla*_NDM_-positive isolates using the microdilution broth method following the Clinical and Laboratory Standards Institute (CLSI) guidelines (38). The MIC results were interpreted according to CLSI documents M100-ED3015 (38) and European Committee on Antimicrobial Susceptibility Testing breakpoints (39). Escherichia coli ATCC 25922 and K. pneumoniae ATCC 13883 were used as quality-control standards.

### Whole-genome sequencing and bioinformatics analysis.

All *bla*_NDM_-positive isolates were selected for whole-genome sequencing (WGS). Total DNA was extracted using a Magen Genomic DNA purification kit (Magen, Guangzhou, China) as per the manufacturer’s instructions. Indexed DNA libraries were prepared using a KAPA Hyper Prep Kit and sequenced on the Illumina HiSeq X 10 platform (Annoroad, Beijing, China). All draft genomes were assembled using SPAdes, version 3.9.0 (40). A K. pneumoniae strain S6 carrying *bla*_NDM_-IncX3 plasmid was selected and sequenced on the MinION platform (Oxford Nanopore Technologies, Oxford, UK). We used Unicycler v.0.4.8-beta (41) to generate genome assemblies combining the Illumina and MinION sequences. Minimum-spanning trees of all *bla*_NDM_-positive isolates were generated in BioNumerics. A phylogenetic tree was produced using snippy (https://github.com/tseemann/snippy) and gubbins (42) using core-genome alignments and was visualized using the online tool, iTOL (43). Phylogenetic trees for K. pneumoniae based on the core-genome sequences of the isolates were structured using Harvest, version 1.1.2 (44). Multilocus sequence typing (MLST), antimicrobial-resistance genes, virulence-associated genes and K (capsule) and O antigen (LPS) serotype prediction were identified using Kleborate (45). Plasmid types of the *bla*_NDM_-carrying contigs were identified using abricate (https://github.com/tseemann/abricate). All contigs from the WGS and MinION sequencing analyses were annotated using prokka (46). Genetic contexts in the different *bla*_NDM_-carrying plasmids were compared using BLAST Ring Image Generator (BRIG) (47). Gcluster (48) was used to visualize and compare the *bla*_NDM_ genome contexts for all genomes. Bandage (49) was used to predicate and visualize the connection of contigs in which IncX3 plasmids located and contigs in which *bla*_NDM_ located.

### Data availability.

All raw data in this study were deposited in GenBank under Bioproject accession number PRJNA750893.
