# Supplementary material for: Clonal and Horizontal Transmission of blaNDM among Klebsiella pneumoniae in Children’s Intensive Care Units
Source: Microbiol Spectr. 2022 Jun 27;10(4):e01574-21. doi: 10.1128/spectrum.01574-21 (PMC9431529; doi:10.1128/spectrum.01574-21)
Supplement: Supplemental file 1 — Supplemental material. Download spectrum.01574-21-s0001.pdf, PDF file, 0.6 MB [file spectrum.01574-21-s0001.pdf]

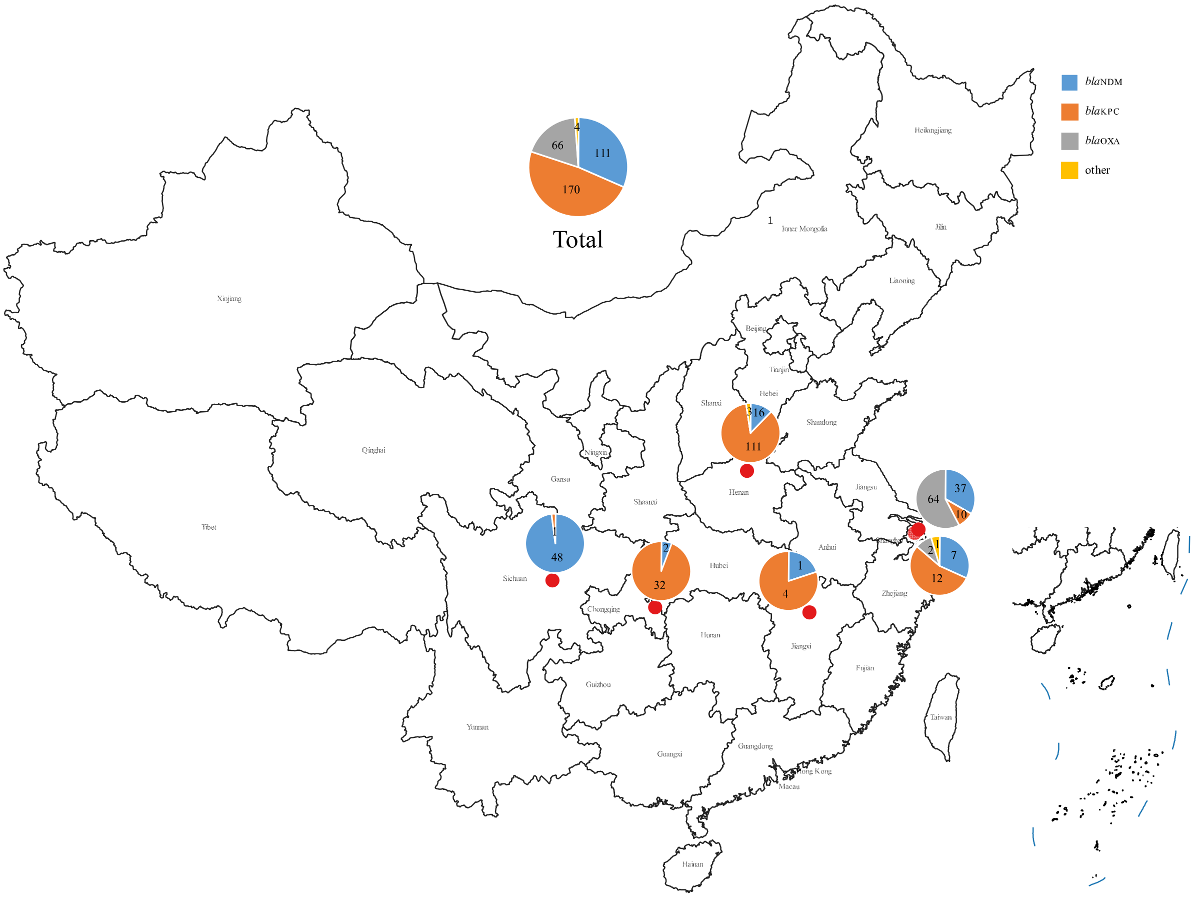

Figure S1. Geographical distribution of CR-KP (n=251) including 111 NDM-KP from six children's hospitals included in the study in China, June 2017–June 2018. Pie charts showing the proportions of different carbapenem-resistance genes in isolates submitted as CR-KP by the children's hospitals.

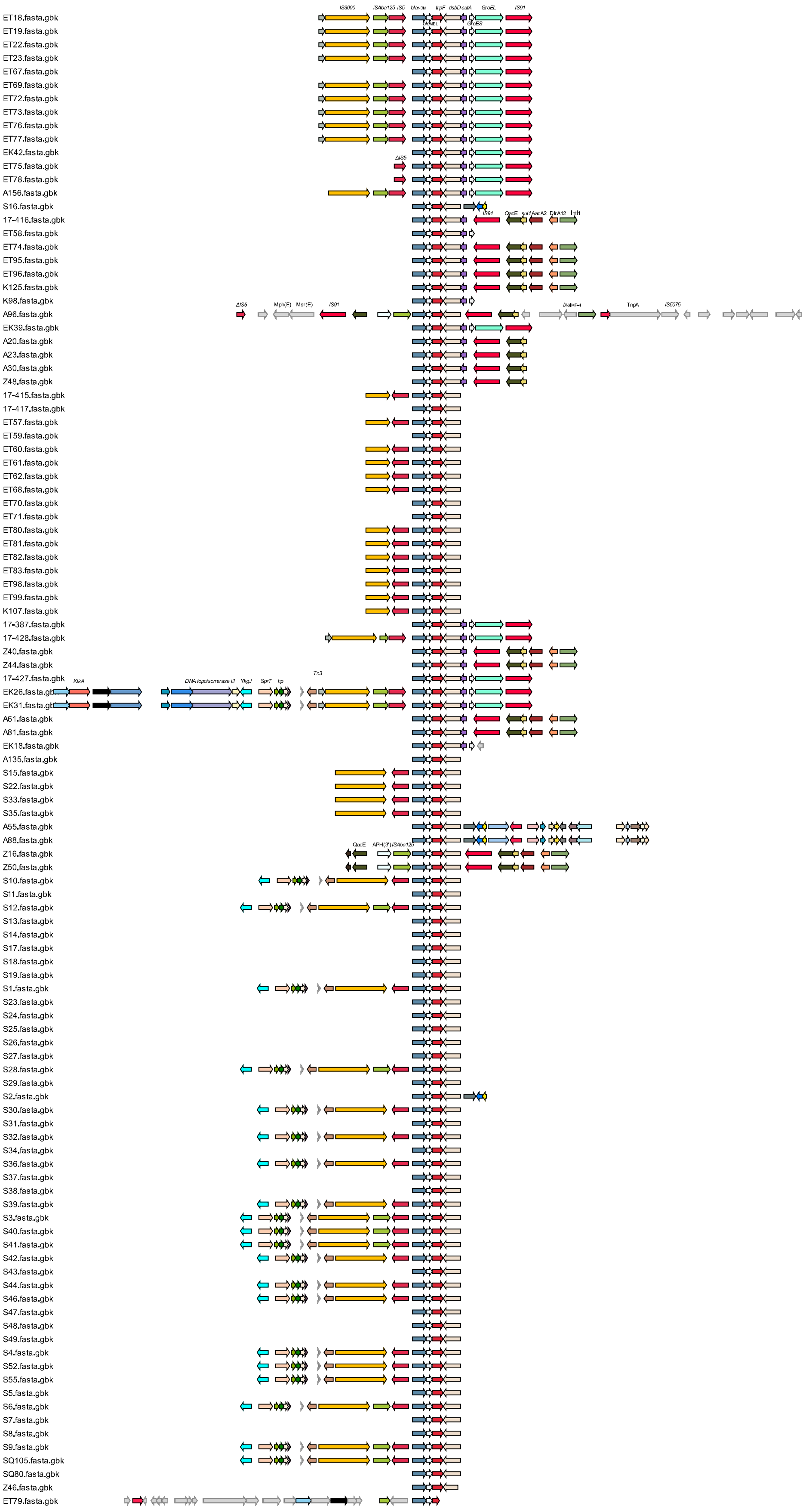

Figure S2. Genetic environment of *bla*NDM in all *K. pneumoniae* isolates. Arrows indicate the direction of gene transcription, and the genes are differentiated by colours.
